# Short-term neurovascular and electrophysiological responses to combined visual and vibration stimulation in older adults with mild cognitive impairment

**DOI:** 10.3389/fnagi.2026.1789422

**Published:** 2026-06-11

**Authors:** Runhong Yao, Kouji Yamada, Takashi Kuremoto, Masahiro Kudo, Fumio Okuyama, Yusuke Morita, Isamu Yanagisawa, Yushi Asaoka, Fumiya Inagaki, Yuki Minegishi, Duojin Wang

**Affiliations:** 1 Faculty of Health Sciences, Nihon Institute of Medical Science, Saitama, Japan; 2 Graduate School of Health Sciences, Fujita Health University, Toyoake, Aichi, Japan; 3 Department of Information Technology and Media Design, Nippon Institute of Technology, Saitama, Japan; 4 Institute of Rehabilitation Engineering and Technology, University of Shanghai for Science and Technology, Shanghai, China

**Keywords:** electroencephalography, gamma oscillations, mild cognitive impairment, multisensory stimulation, near-infrared spectroscopy, neural oscillations, neurovascular coupling, theta oscillations

## Abstract

Mild cognitive impairment (MCI) is associated with age-related alterations in neural oscillatory activity and cerebral hemodynamics, while mobility limitations often restrict participation in conventional exercise-based interventions. This study investigated exploratory, single-arm, within-subject short-term neurovascular and electrophysiological responses to a combined visual and vibration stimulation protocol in older adults with MCI. Cerebral blood flow was assessed using near-infrared spectroscopy (NIRS) in 27 participants, and electroencephalography (EEG) was additionally recorded in a subgroup of nine participants (*n* = 9) drawn from the same cohort. The intervention consisted of simultaneous 40 Hz visual flicker and low-frequency mechanical vibration delivered during a seated condition. Changes in total hemoglobin concentration and EEG power in predefined frequency bands were analyzed before and after the intervention, with additional time-window analyses to examine temporal dynamics. At baseline, NIRS revealed higher total hemoglobin levels in the left prefrontal cortex compared with the right. Following the intervention, total hemoglobin decreased significantly in both hemispheres, with a larger reduction observed in the right hemisphere. Time-resolved analyses demonstrated an earlier hemodynamic response in the right hemisphere, followed by a delayed response in the left. EEG analyses showed a reduction in frontal gamma-band power accompanied by an increase in theta-band power in temporoparietal regions after the intervention. These electrophysiological changes were stable across time windows. Together, these findings suggest that short-term combined visual and vibration stimulation was associated with asymmetric and time-dependent changes in cerebral hemodynamics and neural oscillatory activity in older adults with MCI. This non-volitional stimulation approach may provide a feasible experimental framework for investigating brain responses in aging populations with limited mobility. Given the exploratory, uncontrolled design, acute measurement window, and small EEG subgroup, these findings should be interpreted as hypothesis-generating rather than as evidence of clinical or neuroplastic efficacy.

## 1 Introduction

The prevalence of dementia and Alzheimer’s disease (AD) continues to increase with population aging, creating a growing global public health burden (Prince et al., 2015; Nichols et al., 2022). AD is a progressive neurodegenerative disorder, and current therapeutic options remain limited in their ability to halt or reverse disease progression (Long and Holtzman, 2019; Cummings et al., 2021). As a result, there is sustained interest in non-pharmacological approaches that may support brain function in older adults with cognitive impairment.

Physical activity has been shown to support synaptic plasticity and attenuate age-related cognitive decline (van Praag, 2009; Lourenco et al., 2019). However, individuals with AD or its prodromal stage, mild cognitive impairment (MCI), often experience motor, sensory, or motivational limitations that restrict participation in conventional exercise-based interventions. These constraints highlight the need for alternative, low-burden approaches that can elicit measurable neurophysiological responses without requiring active engagement.

Neural oscillations in distinct frequency bands play fundamental roles in cognition. Gamma-band activity (∼40 Hz) is linked to enhanced neuronal coordination and has been associated with the clearance of pathogenic proteins in animal models (Iaccarino et al., 2016; Adaikkan et al., 2019), while theta-band oscillations (∼4–8 Hz) are central to spatial navigation and memory encoding (Maidenbaum et al., 2018; Kota et al., 2020). Interactions between these rhythms are hypothesized to support cognitive functions such as working memory (Lisman and Jensen, 2013; Igarashi, 2015). Although external rhythmic stimulation can entrain endogenous neural oscillations (Tavano et al., 2022; Saberi and Hickok, 2023), translating these insights into feasible interventions for older adults remains challenging. Crucially, whether combined rhythmic stimulation across sensory modalities produces a detectable physiological response in older adults with MCI is poorly understood.

The present exploratory study, therefore, investigated the short-term neurophysiological effects of a single session using a polyrhythmic transitive motion device (PTMD) in older adults with MCI. The PTMD combines gamma-frequency (40 Hz) visual flicker with theta-frequency mechanical vibration (Yao et al., 2024), delivered in a seated, non-volitional manner. Cerebral hemodynamics were assessed using near-infrared spectroscopy (NIRS), and electroencephalographic (EEG) activity was recorded in a subgroup. The aim was to characterize immediate post-stimulation changes in cerebral blood flow and oscillatory activity, rather than to evaluate therapeutic efficacy. By integrating multimodal physiological assessment, this study seeks to determine if such combined stimulation yields an interpretable neurovascular and electrophysiological signature, thereby providing foundational data to inform future investigations of rhythmic neuromodulation in aging populations with limited mobility.

## 2 Materials and methods

### 2.1 Experimental apparatus

This study investigated the acute neurophysiological after-effects of a PTMD in older adults with MCI. The PTMD integrates gamma-band visual stimulation with theta-frequency mechanical shaking stimulation in a temporally synchronized manner. The combined stimulation paradigm was designed to engage neural activity across multiple frequency bands through visual and somatosensory pathways.

Visual stimulation was delivered using a Gamma Light Therapy GLS-40 device (Gamma Light Therapy LLC, USA), providing a 40 Hz flickering light stimulus with an illuminance of approximately 300 lx at a viewing distance of 30 cm. Mechanical stimulation was delivered via a vibration platform (WBN5020K, Alinco, China) coupled with a FAV5019N motor, producing vertical oscillatory motion with a displacement amplitude of 10.0 ± 0.5 mm at frequencies ranging from 3.8 to 8.8 Hz (230–530 cycles per minute).

Temporal synchronization between visual and mechanical stimuli was controlled by a microcontroller-based system to ensure stable and consistent timing between the two modalities throughout the intervention period.

### 2.2 Participants

Participants were older adults recruited from daycare facilities. MCI was operationally defined according to the core Petersen criteria (Petersen, 2004; Albert et al., 2011), requiring: (i) subjective cognitive concern reported by the participant, a family member, or daycare staff; (ii) objective cognitive impairment documented by an MMSE score of 22–26; (iii) preserved basic activities of daily living (ADL), as confirmed by a Barthel Index score of 100/100 documented in the participants’ daycare care assessment records., indicating full independence in all basic ADL; and (iv) absence of a dementia diagnosis in the medical record.

All participants were able to walk independently (Functional Ambulation Category ≥ 4). We note that this operational definition did not include confirmatory neuropsychological testing (e.g., MoCA, logical memory subtests) or formal structured screening for depression or delirium; accordingly, the cohort should be considered a clinically defined sample rather than a research-diagnostic MCI group meeting the full Petersen criteria.

Exclusion criteria comprised: (i) a documented history of cerebrovascular disease; (ii) severe systemic comorbidities judged by the attending physician to preclude safe participation; (iii) known photosensitivity or a history of seizures; (iv) a current clinical diagnosis of major depressive disorder or delirium as recorded in the daycare medical records.

All participants provided written informed consent prior to participation. Demographic, cognitive, and clinical characteristics of the NIRS cohort (*n* = 27) and the EEG subgroup (n = 9) are summarised in Table 1.

**Table 1:** Demographic and clinical characteristics of participants.

Characteristic	NIRS cohort (*n* = 27)	EEG subgroup (*n* = 9)	Comment
*n*	27	9	*—*
Age, years (mean ± SD)	82.1 ± 3.7 (74–88)	82.8 ± 3.7 (77–88)	
Sex, female—*n* (%)	19 (70.4%)	6 (66.7%)	
Education, years (mean ± SD)	11.4 ± 2.1 (9–16)	11.6 ± 2.4 (9–16)	
MMSE score (mean ± SD)	23.3 ± 1.3 (22–26)	23.0 ± 1.1 (22–25)	*Inclusion: 22–26*
Functional Ambulation Category (mean ± SD)	4.6 ± 0.5 (4–5)	4.4 ± 0.5 (4–5)	*Inclusion: ≥4 (mobility only)*
Barthel Index (mean ± SD)	100 ± 0	100 ± 0	
Comorbidities—*n* (%)			
Musculoskeletal disorders	18 (66.7%)	5 (55.6%)	
Cardiovascular (HTN, CVD)	19 (70.4%)	6 (66.7%)	
Metabolic (DM, dyslipidemia)	15 (55.6%)	5 (55.6%)	
Other chronic conditions	6 (22.2%)	2 (22.2%)	e.g.*, mild renal/hepatic, sensory impairment*
Medications—*n* (%)			
Antihypertensive	18 (66.7%)	6 (66.7%)	
Antidiabetic	5 (18.5%)	2 (22.2%)	
Statin	9 (33.3%)	3 (33.3%)	
CNS-acting drug (sedative, insomnia)	5 (18.5%)	1 (11.1%)	
Exclusion verified—*n* (%)			
Stroke or other major neurological disorders excluded	27 (100.0%)	9 (100.0%)	
Major depressive disorder excluded	27 (100.0%)	9 (100.0%)	
Delirium excluded	27 (100.0%)	9 (100.0%)	

Values are presented as mean ± SD (range) or n (%). MCI, mild cognitive impairment; MMSE, Mini-Mental State Examination; FAC, Functional Ambulation Category; HTN, hypertension; CVD, cardiovascular disease (excluding cerebrovascular events); DM, diabetes mellitus; CNS, central nervous system. The EEG subgroup (*n* = 9) is a subset of the NIRS cohort (*n* = 27). Exclusion verified by record-based screen for neurological disorders, major depressive disorder, and delirium.

### 2.3 Measurement protocols

#### 2.3.1 Cerebral blood flow assessment (NIRS cohort, *n* = 27)

Prefrontal cerebral hemodynamics were assessed using a continuous-wave NIRS system (HOT-2000, NeU Inc., Japan; two channels; sampling rate: 10 Hz). Optodes were positioned over the left and right frontal regions.

The protocol consisted of three phases:

(i) a 3-min baseline recording during seated rest with eyes open;
(ii) a 10-min PTMD intervention; and
(iii) an immediate 3-min post-intervention recording with eyes open.

Changes in oxygenated hemoglobin concentration (Δoxy-Hb) were measured. Total hemoglobin change (ΔHbT), used as an index of cerebral blood volume, was calculated as the sum of Δoxy-Hb and Δdeoxy-Hb. To enhance sensitivity to cortical hemodynamic changes and reduce superficial signal contamination, a differential pathlength approach (SD3cm – SD1cm) was applied.

#### 2.3.2 Neural oscillations (EEG subgroup, *n* = 9)

Neural oscillatory activity was recorded using a wearable EEG system (EMOTIV EPOC X; 14 channels; sampling rate: 256 Hz), with electrodes positioned according to the international 10–20 system.

The EEG protocol included:

(i) a 6-min baseline recording (3 min eyes open, 3 min eyes closed);
(ii) a 10-min PTMD intervention; and
(iii) an immediate 6-min post-intervention recording (3 min eyes open, 3 min eyes closed).
  Mean spectral power was extracted for the theta (4–8 Hz) and gamma (27–45 Hz) frequency bands. Electrodes and frequency bands of interest were selected *a priori* based on their relevance to frontal gamma activity and posterior theta oscillations reported in prior aging and cognitive impairment studies. Gamma-band analyses focused on frontal electrodes (AF3, AF4, F3, F4, F7, F8) during eyes-open conditions, whereas theta-band analyses focused on temporoparietal electrodes (T7, T8, P7, P8) during eyes-closed conditions.

### 2.4 Statistical analysis

All statistical analyses were performed using SPSS Statistics (version 29, IBM Corp., Armonk, NY, USA). Prior to inferential testing, data distributions were evaluated using the Shapiro–Wilk test in combination with visual inspection of histograms and Q–Q plots. All statistical tests were two-tailed, with a significance level set at *p* < 0.05.

#### 2.4.1 NIRS analysis

NIRS–derived hemodynamic measures (ΔHbT) demonstrated clear deviations from normality, including skewed distributions and increased variance. Given the repeated-measures structure and non-normal distribution of these data, non-parametric statistical methods were selected for NIRS analyses. Baseline hemispheric differences and pre- to post-intervention changes were assessed using paired Wilcoxon signed-rank tests. Effect sizes were calculated as standardized rank-based coefficients (r = Z/√N). To examine temporal dynamics across the six consecutive 30-s post-intervention windows, a Friedman test (non-parametric repeated-measures ANOVA equivalent) was conducted for each hemisphere, followed by Bonferroni-corrected pairwise Wilcoxon signed-rank comparisons. Effect sizes (r) are reported to characterize the magnitude of temporal variation where applicable.

#### 2.4.2 EEG analysis

In contrast, EEG band power measures met normality assumptions at the participant level for the predefined frequency bands and electrodes of interest. Accordingly, parametric statistical methods were applied to EEG data. A three-way repeated-measures ANOVA was applied to EEG band power with three within-subject factors: Intervention (pre vs. post), Electrode, and Frequency Band. For the eyes-open analysis of frontal sites, Electrode had six levels (AF3, AF4, F3, F4, F7, F8); for the eyes-closed analysis of temporoparietal sites, Electrode had four levels (T7, T8, P7, P8). Frequency Band was modelled with two levels of primary interest (theta, 4–8 Hz; gamma, 27–45 Hz). Effect sizes were reported as partial eta squared (η^2^p), and *post hoc* comparisons were conducted where appropriate.

**Figure 1:**
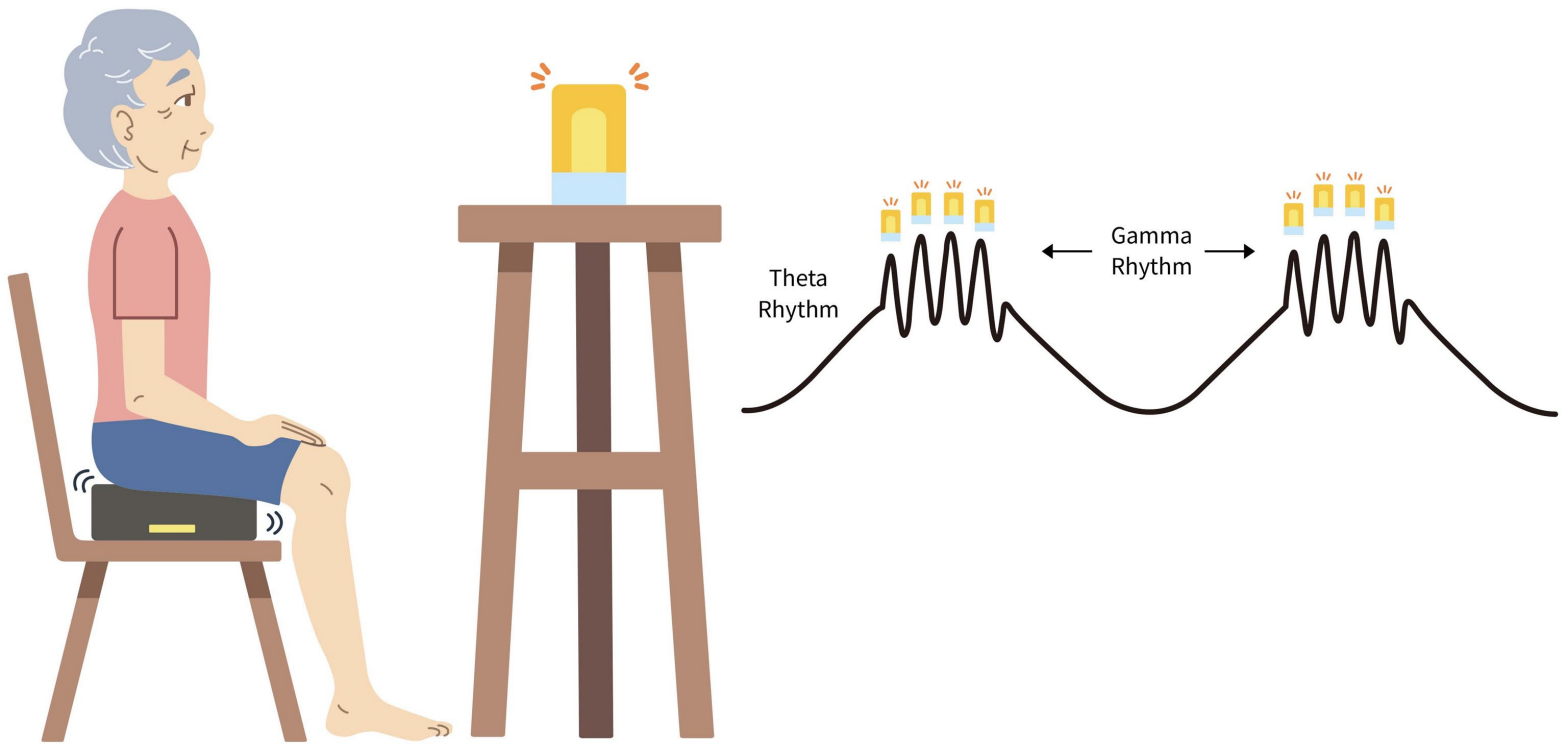
Experimental paradigm and conceptual framework of rhythmic stimulation. **(A)** Illustration of an older adult participant seated on a vibration platform delivering rhythmic mechanical stimulation, synchronized with visual light flicker. The intervention provided simultaneous gamma-frequency visual stimulation and theta-frequency mechanical stimulation in a seated, non-volitional manner. **(B)** Conceptual schematic illustrating the theoretical relationship between theta- and gamma-band neural oscillations, in which faster gamma activity is temporally organized within slower theta rhythms. This schematic represents a conceptual framework motivating the present study and does not imply direct measurement of phase-based cross-frequency coupling.

**Figure 2:**
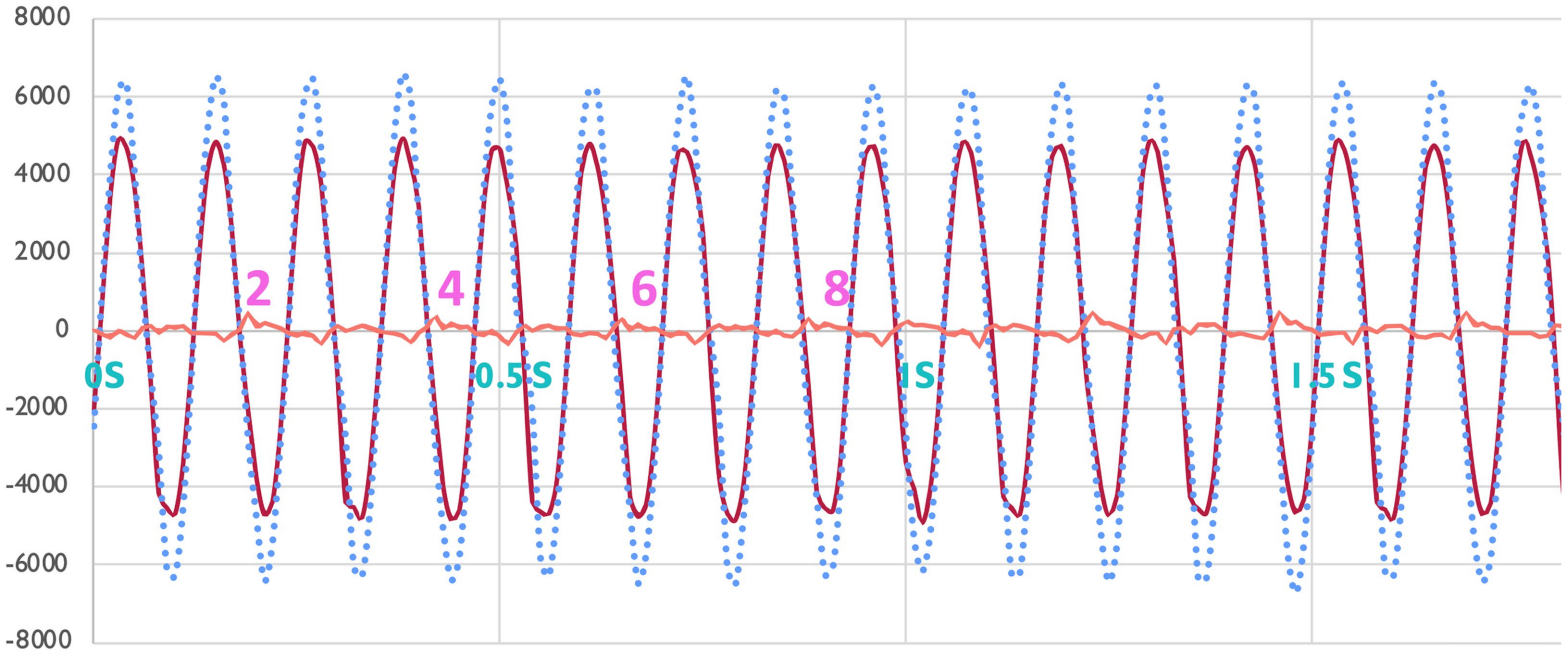
Validation of theta-frequency mechanical vibration during PTMD stimulation. Time-series signals of seat vibration are shown for the *X*-axis (purple), *Y*-axis (blue), and *Z*-axis (orange). The oscillatory motion exhibited a dominant frequency of approximately 8 Hz, confirmed by cycle counts and timestamps (cyan: seconds; magenta: cycles). The *X*-axis component showed the largest initial amplitude, which gradually decreased over time, whereas the *Y*-axis component increased. A minor *Z*-axis component was also observed, indicating multidirectional but predominantly *X*–*Y*–oriented vibration.

Given the exploratory nature of the EEG analysis and the limited sample size (*n* = 9), statistical results are interpreted primarily to describe within-subject response patterns rather than to support population-level inference. No phase-based analyses of cross-frequency coupling (e.g., theta–gamma phase–amplitude coupling) were performed in the present study because the number of electrodes, the sampling rate, and the sample size of the EEG subgroup were all judged to be insufficient for reliable phase-domain inference; such analyses are explicitly deferred to future work (see Figures 1, 2).

### 2.5 Ethical statement

This study was conducted in accordance with the Declaration of Helsinki and approved by the Ethics Committee of Japan University of Health Sciences (Approval No. P2103). Written informed consent was obtained from all participants prior to enrollment. Study procedures and potential risks were explained to participants and, where applicable, their legal guardians.

## 3 Results

### 3.1 Cerebral blood flow (NIRS data)

#### 3.1.1 Baseline hemispheric distribution

**Figure 3:**
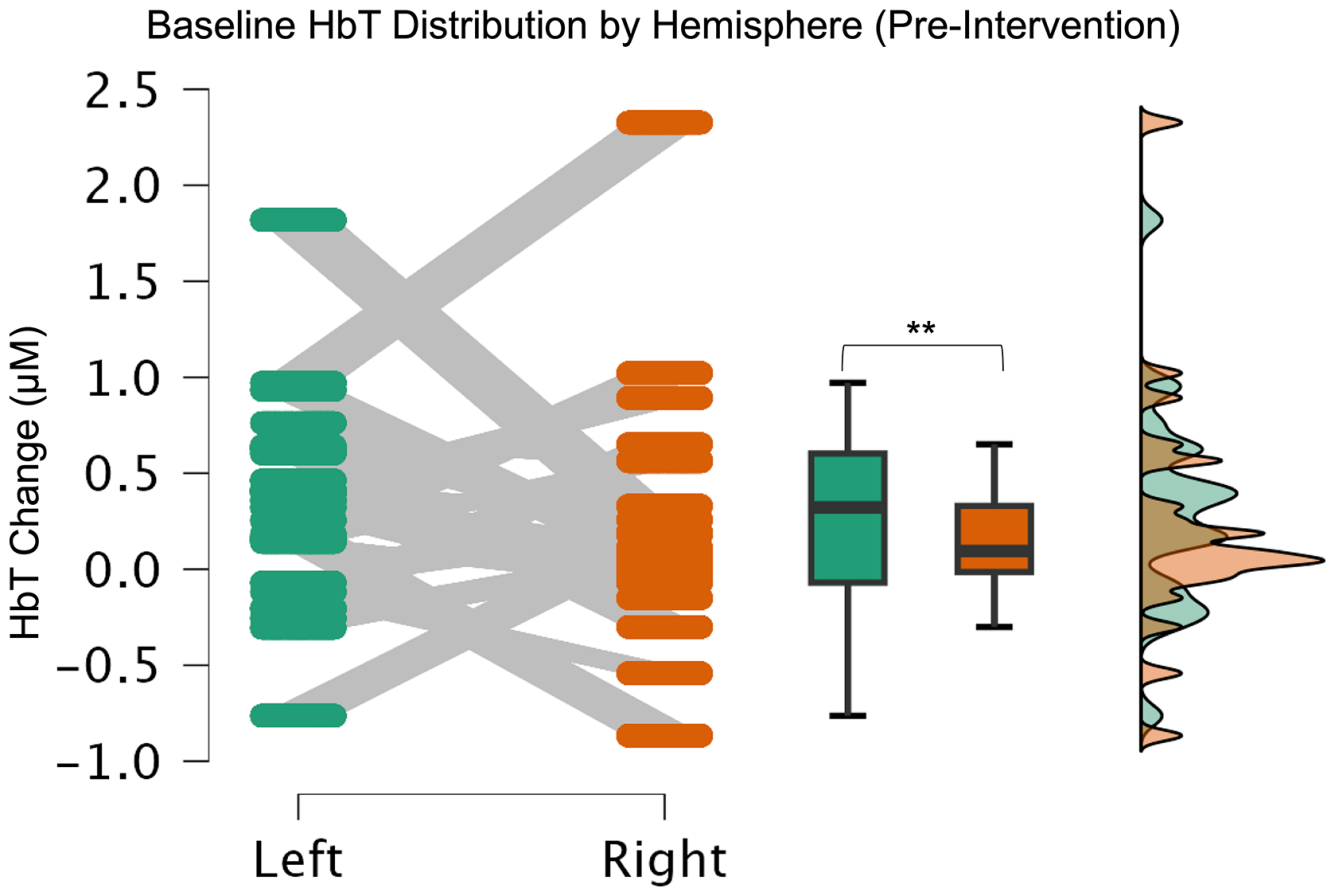
Baseline hemispheric comparison of total hemoglobin (HbT) in frontal regions. Mean total hemoglobin (HbT) values prior to PTMD intervention were significantly higher in the left frontal region (0.308 ± 0.508 μM) than in the right frontal region (0.225 ± 0.584 μM; Wilcoxon signed-rank test: *Z* = −26.166, *p* < 0.001). Error bars indicate standard deviation.

At baseline, total hemoglobin (HbT) values exhibited a consistent hemispheric asymmetry across participants. Time-averaged HbT was higher in the left frontal region (0.308 ± 0.508 μM) than in the right frontal region (0.225 ± 0.584 μM). A paired Wilcoxon signed-rank test confirmed a significant leftward shift in baseline HbT values (*Z* = −26.166, *p* < 0.001, *r* = 0.39), indicating asymmetric prefrontal hemodynamic distribution prior to intervention (Figure 3).

#### 3.1.2 Pre- to post-intervention HbT changes

**Figure 4:**
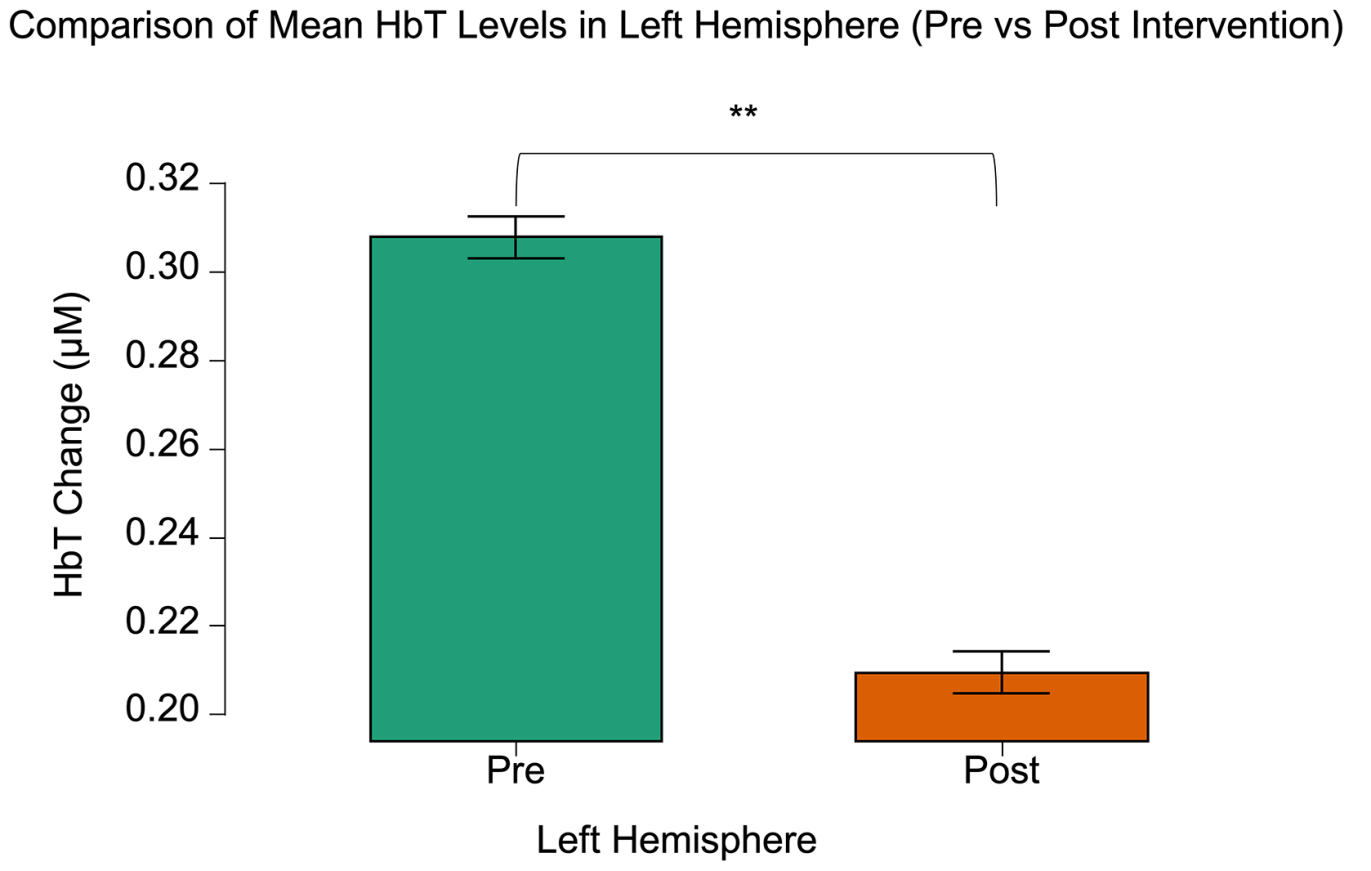
Pre- to post-intervention changes in left frontal HbT. HbT in the left frontal region decreased significantly following the PTMD intervention, from 0.308 ± 0.508 μM at baseline to 0.209 ± 0.543 μM post-intervention (Wilcoxon signed-rank test: *Z* = −21.278, *p* < 0.001). Error bars indicate standard deviation.

Following the PTMD intervention, HbT values decreased in both hemispheres. In the left frontal region, HbT declined from 0.308 ± 0.508 μM at baseline to 0.209 ± 0.543 μM (*Z* = −21.278, *p* < 0.001; *r* = 0.32), with 51% of participants showing an average post-intervention reduction (Figure 4).

**Figure 5:**
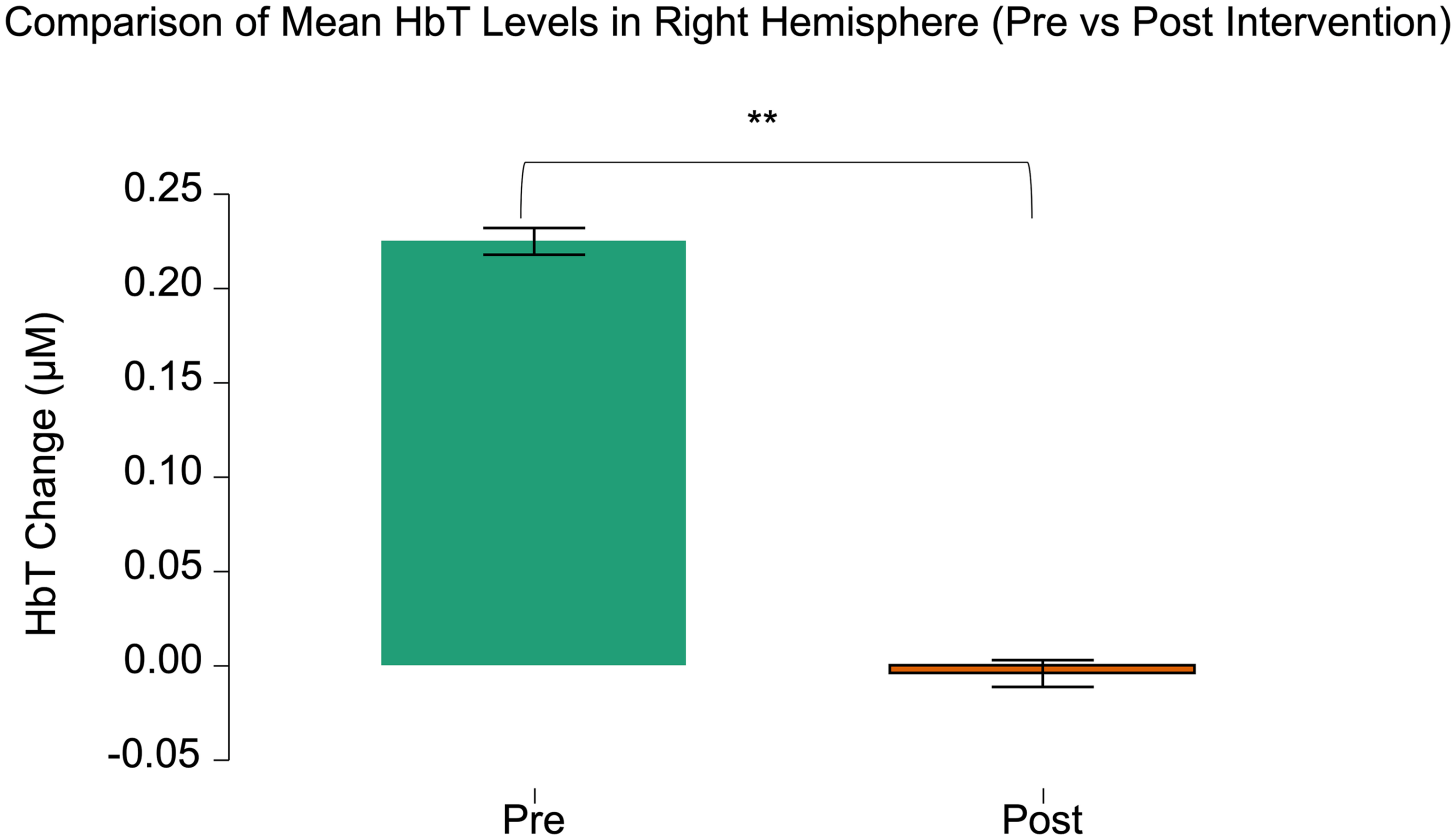
Pre- to post-intervention changes in right frontal HbT. Right frontal HbT decreased from 0.225 ± 0.584 μM at baseline to −0.004 ± 0.871 μM following the PTMD intervention (Wilcoxon signed-rank test: *Z* = −25.671, *p* < 0.001). The magnitude of reduction was larger than that observed in the left frontal region. Error bars indicate standard deviation.

In the right frontal region, a larger downward shift was observed, with HbT decreasing from 0.225 ± 0.584 μM to −0.004 ± 0.871 μM (*Z* = −25.671, *p* < 0.001; *r* = 0.38) (Figure 5). The magnitude of reduction was greater on the right side than on the left, indicating hemispheric differences in post-intervention hemodynamic response.

#### 3.1.3 Temporal profile of post-intervention HbT

**Figure 6:**
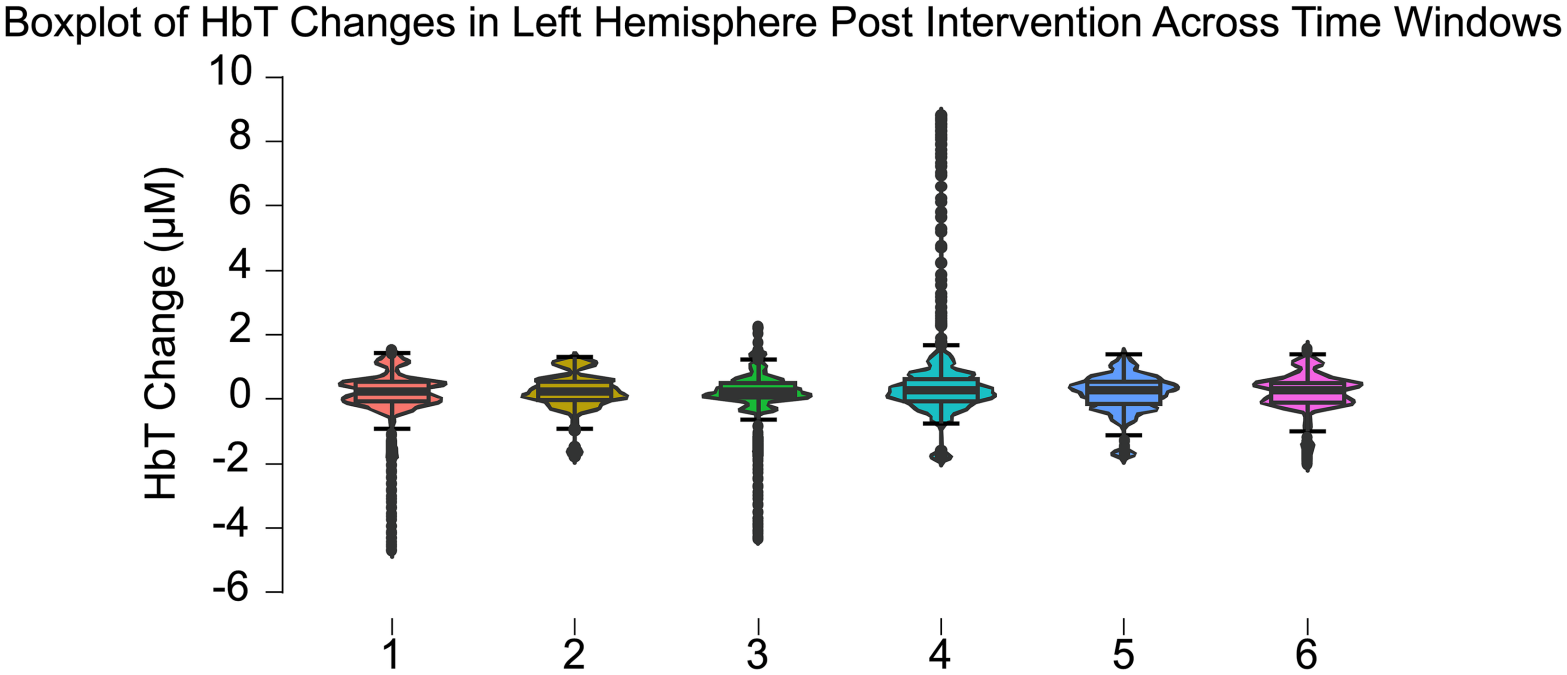
Time-window analysis of post-intervention HbT in the left frontal region (eyes open). Post-intervention HbT values were segmented into six consecutive 30 s windows. HbT reached a relative maximum during the fourth window (90–120 s; 0.259 ± 0.791 μM) and declined in the subsequent window (120–150 s; 0.171 ± 0.599 μM). Bonferroni-corrected comparisons indicated that the fourth window differed significantly from the other windows (*p* < 0.001). Error bars indicate standard deviation.

To characterize post-intervention dynamics, HbT values during the eyes-open post-intervention period were segmented into six consecutive 30-s time windows. In the left hemisphere, HbT reached a relative maximum during the fourth window (90–120 s; 0.259 ± 0.791 μM) and subsequently declined, reaching a minimum in the fifth window (120–150 s; 0.171 ± 0.599 μM) (Figure 6).

**Figure 7:**
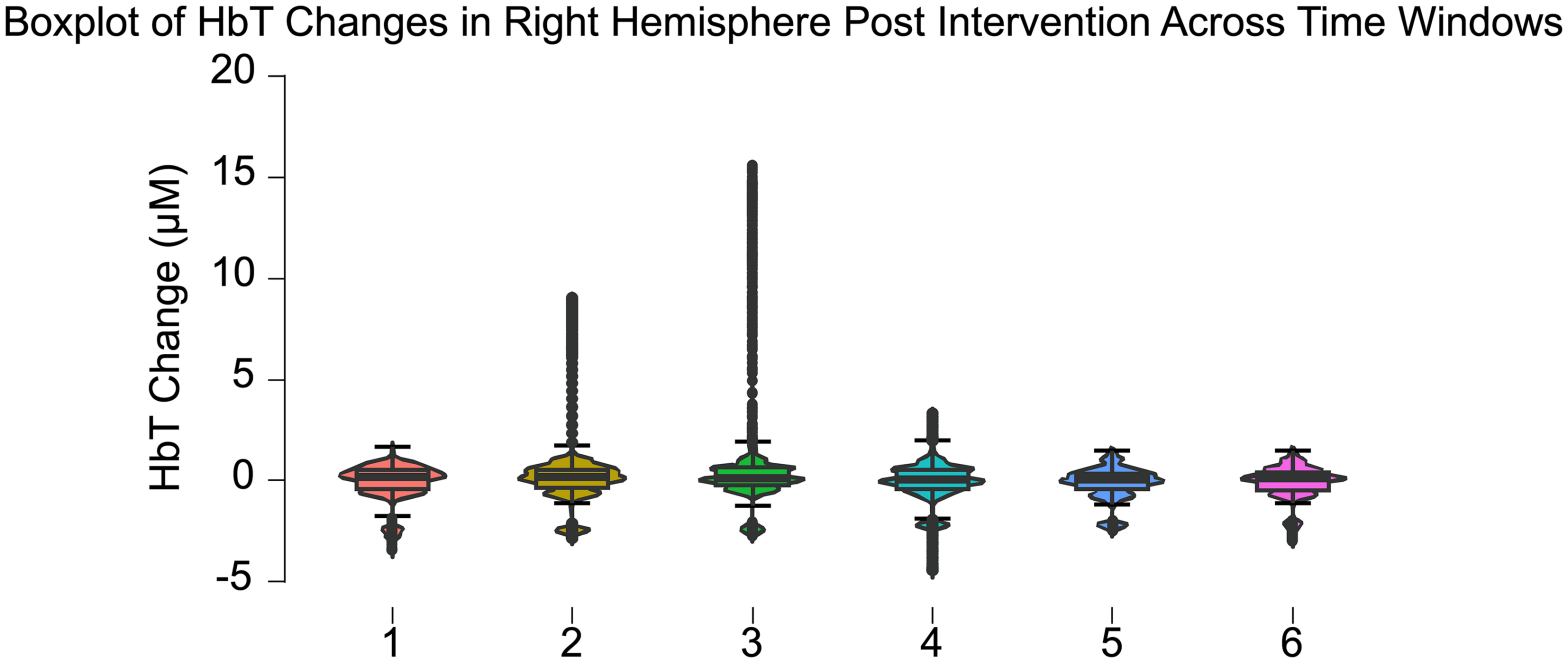
Time-window analysis of post-intervention HbT in the right frontal region (eyes open). A transient HbT peak was observed in the third window (60–90 s; 0.305 ± 1.923 μM), followed by a marked decrease in the fifth window (−0.123 ± 0.802 μM). Temporal variability across windows was greater in the right frontal region than in the left. Error bars indicate standard deviation.

In contrast, the right hemisphere exhibited an earlier transient increase, with HbT peaking in the third window (60–90 s; 0.305 ± 1.923 μM), followed by a marked decrease in the fifth window (−0.123 ± 0.802 μM) (Figure 7). The overall temporal variability was greater in the right hemisphere (SD = 1.168 μM) than in the left hemisphere (SD = 0.630 μM), indicating increased temporal instability of right frontal hemodynamic responses following stimulation.

### 3.2 EEG findings (EMOTIV data)

Given the exploratory nature of the EEG analysis and the limited sample size (*n* = 9), EEG results are reported to describe within-subject response patterns rather than to support population-level inference.

#### 3.2.1 Frontopolar and frontal electrodes: gamma-band activity (AF3, AF4, F3, F4, F7, F8; eyes-open)

EEG band power data satisfied normality assumptions, and parametric analyses were applied. Repeated-measures ANOVA revealed significant main effects of intervention condition (*F* = 7.587, *p* = 0.043), electrode location (*F* = 7.500, *p* = 0.003), and frequency band (*F* = 118.772, *p* < 0.001). A significant Intervention × Frequency Band interaction was also observed (*F* = 254.231, *p* < 0.001), indicating frequency-dependent modulation following intervention.

**Figure 8:**
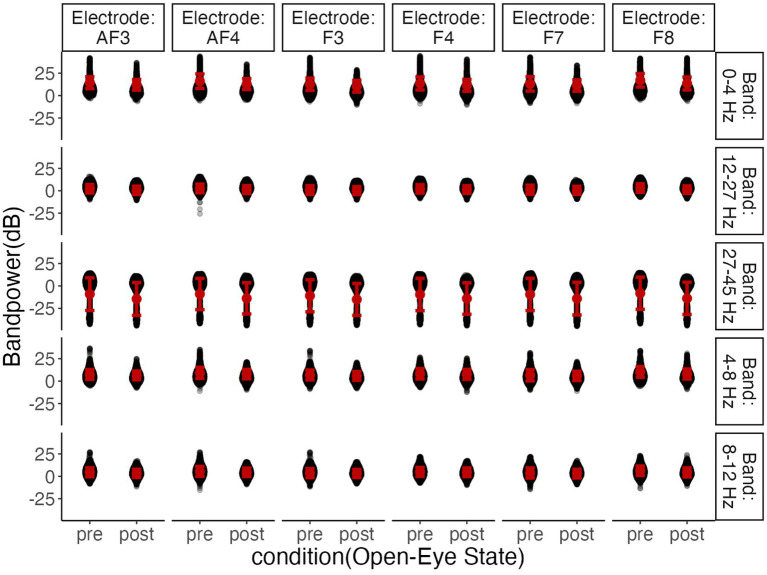
Post-intervention changes in gamma-band power at frontal electrodes (eyes open). Gamma-band (27–45 Hz) power decreased following the intervention across frontal electrodes (AF3, AF4, F3, F4, F7, F8), with the largest reductions observed at AF4 (−48.1%) and F4 (−54%). Repeated-measures ANOVA revealed a significant main effect of frequency band (*F* = 3247.811, *p* < 0.001). In contrast, theta-band (4–8 Hz) power showed a modest increase.

Post-hoc analyses focusing on the gamma band (27–45 Hz) demonstrated a pronounced reduction in gamma power at the right frontal site AF4, decreasing from 4.879 ± 0.062 dB pre-intervention to 2.531 ± 0.057 dB post-intervention, corresponding to a 48.1% reduction. The magnitude of this change was large (Cohen’s d = 1.21). Across frontal electrodes, post-intervention power decreased in all analyzed frequency bands except for the theta band (4–8 Hz), which showed an increase (Figure 8).

#### 3.2.2 Temporal and parietal electrodes: theta- and gamma-band activity (P7, P8, T7, T8; eyes-closed)

**Figure 9:**
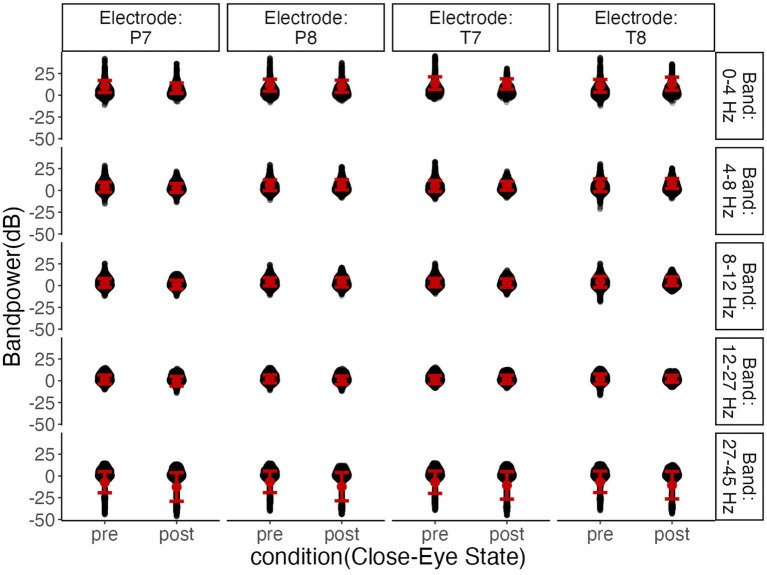
Post-intervention theta- and gamma-band power changes at temporoparietal electrodes (eyes closed). Following the PTMD intervention, theta-band power increased significantly at the right temporal electrode T8 (+23%, *p* < 0.001), whereas gamma-band power decreased at the right parietal electrode P8 (−12.306 dB, Cohen’s *d* = 1.47). These findings indicate concurrent, frequency-specific modulation of theta and gamma activity at temporoparietal sites.

Analysis of temporal and parietal electrodes revealed significant effects involving frequency band and intervention (all *p* < 0.01; detailed statistics are provided in Supplementary Table S1). Following the intervention, theta-band power increased significantly at the right parietal electrode P8 (*Δ* + 6.462 dB, *p* < 0.001). In contrast, gamma-band power at the same electrode showed a significant reduction (Δ − 12.306 dB, *p* < 0.001), with a large effect size (Cohen’s d = 1.47) (Figure 9).

#### 3.2.3 Temporal stability of EEG power

Across six consecutive 30-s post-intervention windows, EEG band power exhibited minimal variation, with changes generally below 1% and small effect sizes (partial η^2^ < 0.02). At electrode F4, a modest decrease was observed during the final window (150–180 s), whereas sustained gamma suppression was observed at AF4 throughout the post-intervention period, indicating relative temporal stability of the observed spectral changes.

### 3.3 Summary of results

Overall, the PTMD intervention was associated with asymmetric frontal hemodynamic responses, characterized by greater temporal variability and larger post-intervention reductions in HbT in the right hemisphere compared with the left. EEG recordings demonstrated frequency-specific modulation, with widespread gamma-band suppression occurring alongside theta-band enhancement, particularly in temporal–parietal regions. Time-resolved analyses indicated a phased response profile, consisting of an early right-hemisphere hemodynamic fluctuation (60–90 s) followed by a delayed left-hemisphere response (90–120 s). Together, these findings demonstrate that short-term combined stimulation produces a detectable and temporally structured neurophysiological signature in older adults with MCI.

## 4 Discussion

### 4.1 Asymmetric neurovascular responses in MCI

The asymmetric response between the left and right frontal lobes observed in this study highlights a potential hemodynamic signature of the MCI stage. Baseline total hemoglobin (HbT) was significantly higher in the left frontal lobe compared to the right. This left-dominant pattern contrasts with the typical right hemisphere dominance reported in patients with AD (Liu et al., 2018; Vogel et al., 2021), and may reflect a stage-specific shift in prefrontal resource allocation during MCI, consistent with reports of altered hemispheric metabolism in prodromal stages (Yu et al., 2020; Osei et al., 2025). Following the PTMD intervention, a more pronounced decrease in HbT was observed in the right hemisphere compared to the left, suggesting differential hemispheric sensitivity to the external rhythmic stimulation.

### 4.2 Temporally phased neurovascular and oscillatory dynamics

Time-resolved analysis revealed distinct, phased patterns across modalities and hemispheres, delineating the short-term after-effects of the intervention.

(i) Early Post-Intervention Phase (0–90 s): In the right hemisphere, a sharp hemodynamic peak was observed in the third time window. This coincided electrophysiologically with marked suppression of gamma-band power at right frontal sites and a concurrent increase in theta-band power at right temporal sites, suggesting a rapid, frequency-specific reconfiguration of oscillatory activity following stimulation.
(ii) Middle Post-Intervention Phase (90–150 s): The left hemisphere exhibited a delayed increase in HbT, peaking in the fourth window (90–120 s; +0.259 ± 0.791 μM), without a corresponding pronounced shift in local EEG power.
(ii) Late Post-Intervention Phase (150–180 s): Fluctuations in both hemodynamic (HbT ± 0.05 μM) and oscillatory (EEG power variation <1%) signals were minimized, suggesting a return toward a stabilized physiological state.

These temporally offset responses suggest that a brief PTMD intervention was associated with a cascade of physiological after-effects, a phenomenon observed in other forms of sensory and cognitive neuromodulation (Stefan et al., 2008), beginning with right-hemisphere–biased responses and followed by a delayed left-hemisphere vascular response.

### 4.3 Frequency-specific and coordinated oscillatory modulation

EEG analyses revealed frequency- and region-dependent after-effects. The most consistent finding was a reciprocal pattern: a decrease in gamma-band power (e.g., at parietal site P8: *Δ* − 12.306 dB, *d* = 1.47) alongside an increase in theta-band power (at P8: Δ + 6.462 dB). This reciprocal pattern is consistent with a coordinated, frequency-specific modulation rather than a global shift in brain activity. While this pattern is consistent with the theoretical framework of theta-gamma cross-frequency interactions (Aron and Yankner, 2016), the present study did not perform phase-amplitude coupling (PAC) analysis; these findings therefore represent correlated changes in band-limited power and should not be interpreted as direct evidence of cross-frequency coupling. Formal phase-based analysis is deferred to future work employing research-grade EEG with higher electrode density.

### 4.4 Relationship between oscillatory and hemodynamic after-effects

The concurrent measurement of EEG and NIRS allowed for an integrated view of the post-intervention state. The two hemispheres showed qualitatively different neurovascular–oscillatory profiles. In the right hemisphere, the decrease in gamma-band power co-occurred with a decrease in HbT, consistent with a coupled reduction in local neural activity and metabolic demand predicted by neurovascular coupling principles (Aubert et al., 2001; Aubert and Costalat, 2002). In contrast, the left hemisphere exhibited a delayed HbT rise without a corresponding local EEG power change, suggesting a temporally dissociated vascular response. This spatial dissociation raises the possibility of regional differences in neurovascular coupling efficiency, which has been shown to vary across cortical regions and in aging (Yabluchanskiy et al., 2021; Bennett et al., 2024; Ishikawa et al., 2025), or differing temporal profiles of vascular and electrical responses to the intervention.

### 4.5 Interpretation within context and limitations

The observed patterns—including left-dominant baseline asymmetry, a right-hemisphere–biased acute response, and reciprocal modulation of gamma- and theta-band activity—together tentatively describe a distinct physiological response profile to multi-sensory rhythmic stimulation in individuals with MCI. These findings are broadly consistent with established concepts, such as compensatory prefrontal recruitment in aging, the involvement of theta oscillations in memory-related networks, and the hypersynchrony hypothesis proposed in AD (Supp et al., 2011; Verret et al., 2012; Wang et al., 2017; Prabhu et al., 2024). The principal limitations of this exploratory study are summarized as follows:

(1) Single-arm design with no active control or sham condition: pre-to-post changes cannot be causally attributed to the stimulation itself rather than to time, repeated measurement, or non-specific effects.
(2) Single-session, acute-measurement design: only post-intervention effects within a brief quiet rest window were captured; whether repeated sessions produce cumulative or sustained changes is untested.
(3) Small EEG subgroup (*n* = 9): all EEG findings are exploratory and describe within-subject response patterns rather than supporting population-level inference.
(4) Consumer-grade wearable EEG (EMOTIV EPOC X, 14 channels, 256 Hz): limited spatial coverage and restricted high-frequency bandwidth preclude precise cortical localisation; gamma-band effects should be interpreted as relative within-subject modulations.
(4) No phase-based cross-frequency coupling (PAC) analysis: observed reciprocal gamma and theta changes represent correlated band-limited modulations and are not direct evidence of PAC.
(5) Clinically defined MCI cohort classified by MMSE only, without MoCA or structured screening for depression/delirium (SCID-5, CAM).
(6) No behavioural, neuropsychological, or longitudinal outcome measures were obtained: the study cannot speak to clinical efficacy, cognitive benefit, or neuroplastic change; all interpretive claims are restricted to acute physiological after-effects.
(7) Laterality findings are exploratory observations consistent with prefrontal asymmetry literature in aging and require replication in pre-registered, controlled studies before any mechanistic claim is endorsed.

### 4.6 Future directions

Future studies should build on these findings by employing research-grade EEG and multi-channel NIRS to examine both real-time and post-stimulation dynamics with robust artifact handling. Direct phase-based coupling analyses will be necessary to test hypothesized cross-frequency mechanisms. Finally, controlled longitudinal designs incorporating behavioral and cognitive outcomes are required to determine whether these acute physiological responses translate into sustained functional benefits for individuals with MCI.

## 5 Conclusion

This pilot study suggests that a brief session of combined gamma-frequency visual stimulation and theta-frequency vibratory stimulation is associated with rapid, measurable changes in cerebral hemodynamics and neural oscillatory activity in older adults with MCI. The principal observations include: (1) a baseline left-dominant prefrontal hemodynamic asymmetry that changes following the intervention; (2) a temporally ordered response pattern characterized by right-hemisphere–biased hemodynamic and oscillatory changes followed by a delayed left frontal vascular response; and (3) coordinated, frequency-specific modulation of neural oscillations, with reductions in gamma-band power occurring alongside increases in theta-band power in distinct cortical regions.

Collectively, these findings describe a short-term physiological response profile following multisensory rhythmic stimulation in individuals with MCI. The results indicate that non-invasive, dual-frequency stimulation is associated with spatially and temporally specific alterations in neurovascular and electrophysiological measures. Based on these observations, a testable hypothesis is generated that hemispheric asymmetry and phased response patterns may reflect compensatory or adaptive processes operating during the MCI stage; however, this interpretation requires direct validation in future studies.

Importantly, the present study was not designed to assess cognitive performance, clinical outcomes, or long-term effects. Accordingly, the findings should be interpreted as characterizing acute neurophysiological after-effects rather than as evidence of therapeutic efficacy or neuroplastic adaptation. Nonetheless, these results provide a methodological foundation and physiological rationale for future controlled and longitudinal investigations incorporating behavioral outcomes, appropriate comparison groups, and advanced signal analyses to determine whether such stimulation paradigms have potential relevance for supporting cognitive health in aging populations.

## Data Availability

Publicly available datasets were analyzed in this study. This data can be found at https://data.mendeley.com/datasets/w8h3f58bbm/2, DOI: 10.17632/w8h3f58bbm.2.
